# Supplementary material for: Analysis of IMRT patient specific quality assurance using EPID measurement data from the Halcyon linear accelerator
Source: PLoS One. 2026 May 14;21(5):e0348615. doi: 10.1371/journal.pone.0348615 (PMC13175351; doi:10.1371/journal.pone.0348615)
Supplement: Attachment — Submitted filename: Response to Reviewers.docx [file pone.0348615.s008.docx]

Department of Radiation Oncology

Chonnam National University Medical School

160 Baekseo-Ro Dong-Gu

Gwangju, 61469, Korea

Tel)82-61-379-7225, Fax)82-61-379-7249

April 8, 2026

Editor-in-Chief

PLOS ONE

Dear Editor and Reviewers,

Thank you very much for providing the review results and for your valuable comments on our manuscript. We sincerely appreciate the time and effort devoted to evaluating our work.

Below are our point-by-point responses to the reviewers' comments.

Reviewer #1: This study is an important investigation about the patient specific quality assurance using EPID data from Halcyon Linac. As the clinical usage of these comparatively new Linacs are increasing, this paper is practically important. The authors have covered all the necessary points aimed for this investigation. Data and the statistical analysis clearly support the conclusion s. Authors made the data fully available without restrictions. The manuscript is well structured and fits the guideline of the journal. If the figures meet the criteria, this manuscript is acceptable for publication.

**Thank you for your positive evaluation and for the favorable decision regarding the publication of our manuscript. We have thoroughly reviewed the journal’s guidelines for figure files and have resubmitted the revised versions to ensure full compliance. We sincerely appreciate your constructive feedback and support.**

Reviewer #2: The manuscript is technically sound, well written and easy to understand.

The topic is highly relevant to PSQA and clearly demonstrates the robustness of EPID based dosimetry. (We have seen similar results in our department).

The axis titles in Figure 3 are hard to read.

The bar graph titles in Figures 4 & 5 are too small to read.

**Thank you for your valuable review and suggestions. As requested, we have revised the figure files to improve their clarity and readability. The specific modifications are as follows:**

**- Figure 3: The font size of the axis titles has been increased and bolded to enhance legibility.**

**- Figures 4 & 5: The titles of the bar graphs have been made larger and bolder to ensure they are easier**

**to read.**

**We have resubmitted these updated files for your reconsideration.**

We would like to express our gratitude once again for your valuable comments. We hope that our revisions adequately address the concerns raised and will be favorably considered during the re-review process.

Sincerely,

Ju-Young Song. Ph.D.

E-mail: jysong@jnu.ac.kr
